# Circulating Biomarkers of Cell Adhesion Predict Clinical Outcome in Patients with Chronic Heart Failure

**DOI:** 10.3390/jcm9010195

**Published:** 2020-01-10

**Authors:** Elke Bouwens, Victor J. van den Berg, K. Martijn Akkerhuis, Sara J. Baart, Kadir Caliskan, Jasper J. Brugts, Henk Mouthaan, Jan van Ramshorst, Tjeerd Germans, Victor A. W. M. Umans, Eric Boersma, Isabella Kardys

**Affiliations:** 1 Erasmus MC, 3000CA Rotterdam, The Netherlands; 2 Olink Proteomics, SE-751 83 Uppsala, Sweden; 3 Northwest Clinics, 1815JD Alkmaar, The Netherlands

**Keywords:** biomarkers, cell adhesion molecule, heart failure, repeated measurements

## Abstract

Cardiovascular inflammation and vascular endothelial dysfunction are involved in chronic heart failure (CHF), and cellular adhesion molecules are considered to play a key role in these mechanisms. We evaluated temporal patterns of 12 blood biomarkers of cell adhesion in patients with CHF. In 263 ambulant patients, serial, tri-monthly blood samples were collected during a median follow-up of 2.2 (1.4–2.5) years. The primary endpoint (PE) was a composite of cardiovascular mortality, HF hospitalization, heart transplantation and implantation of a left ventricular assist device and was reached in 70 patients. We selected the baseline blood samples in all patients, the two samples closest to a PE, or, for event-free patients, the last sample available. In these 567 samples, associations between biomarkers and PE were investigated by joint modelling. The median age was 68 (59–76) years, with 72% men and 74% New York Heart Association class I–II. Repeatedly measured levels of Complement component C1q receptor (C1qR), Cadherin 5 (CDH5), Chitinase-3-like protein 1 (CHI3L1), Ephrin type-B receptor 4 (EPHB4), Intercellular adhesion molecule-2 (ICAM-2) and Junctional adhesion molecule A (JAM-A) were independently associated with the PE. Their rates of change also predicted clinical outcome. Level of CHI3L1 was numerically the strongest predictor with a hazard ratio (HR) (95% confidence interval) of 2.27 (1.66–3.16) per SD difference in level, followed by JAM-A (2.10, 1.42–3.23) and C1qR (1.90, 1.36–2.72), adjusted for clinical characteristics. In conclusion, temporal patterns of C1qR, CDH5, CHI3L1, EPHB4, ICAM2 and JAM-A are strongly and independently associated with clinical outcome in CHF patients.

## 1. Introduction

In recent decades, chronic heart failure (CHF) has emerged as a complex syndrome that involves a broad array of biological pathways [1,2]. In this context, CHF has been associated with endothelial dysfunction and low-grade inflammation [3]. Moreover, the role of the immune system in the development and progression of CHF has received considerable attention in recent years [4]. An essential step in this process is the adherence of circulating mononuclear cells to the vascular endothelium through binding of cell adhesion molecules (CAMs) that are expressed on the surface of these mononuclear cells, or on the endothelial cells, or on both [5]. Binding of the mononuclear cells to the endothelium leads to extravasation of these cells into the involved tissue [5], promoting structural deterioration, which eventually contributes to reduced cardiac function. Interestingly, enhanced expression of CAMs has been found within the myocardial microvasculature of patients with severe CHF as compared to healthy subjects [6], providing further support that vascular inflammation might be involved in the propagation and progression of CHF.

Different classes of CAMs have been identified, and among them are selectins, integrins, cadherins and the immunoglobulin superfamily [7]. In addition, several other molecules are involved in the cell adhesion processes. In more detail, selectins such as platelet (P)-selectin (SELP) are involved in the adhesion of leucocytes to activated endothelium and are known for the typical “rolling” of leucocytes on the surface of the endothelium. Other selectins such as endothelial (E)-selectin (SELE) are involved in the cell extravasation process. Integrins mediate the leucocyte adherence to the vascular endothelium and other cell–cell interactions [8]. Cadherins are an important family of calcium dependent cell–cell adhesion molecules. In addition to their structural role, they have been implicated in the regulation of signaling events [7]. For example, cadherin 5 (CDH5) is a major cell–cell adhesion molecule that forms adherens junctions [9]. Lastly, the immunoglobulin superfamily comprises a diverse group of proteins including intracellular adhesion molecule-1 (ICAM-1), ICAM-2 and ICAM-3, vascular adhesion molecule-1 (VCAM-1), platelet endothelial cell adhesion molecule 1 (PECAM-1) and others, which are expressed on the surface of the endothelial cells and are known for firm adhesion of leucocytes and transendothelial migration [10].

Shedding of CAMs from the cell surface results in measurable levels in peripheral blood [11], which can reflect overexpression of their membrane-bound forms. Since CAMs may thus reflect processes involved in CHF, the association of these circulating biomarkers with clinical outcome provokes interest. Temporal patterns of biomarkers of cell adhesion in CHF, and their associations with an adverse disease course, have not yet been examined. Therefore, in this study, we investigated 12 cell adhesion-related biomarkers repeatedly measured with the Olink Multiplex panel, which contains 92 known human cardiovascular biomarkers that have previously been extensively investigated in the literature as well as exploratory candidates that are thought to carry potential as new biomarkers. Specifically, here, we examined biomarkers from this panel related to the above-described mechanisms (SELP, SELE, CDH5, ICAM-2, and PECAM-1) and other potentially interesting biomarkers related to cell adhesion processes (complement component C1q receptor (C1qR), chitinase-3-like protein 1 (CHI3L1), contactin-1 (CNTN1), ephrin type-B receptor 4 (EPHB4), epithelial cell adhesion molecule (Ep-CAM), integrin beta-2 (ITGB2), and junctional adhesion molecule A (JAM-A). The aim of the present study was to evaluate the association between temporal patterns of these biomarkers of cell adhesion and clinical outcomes in stable patients with CHF.

## 2. Methods

### 2.1. Patient Selection 

A total of 263 patients enrolled in the ‘Serial Biomarker Measurements and New Echocardiographic Techniques in Chronic Heart Failure Patients Result in Tailored Prediction of Prognosis’ (Bio-SHiFT) study were included in the Netherlands. The Bio-SHiFT study is a prospective, observational cohort study of stable patients with CHF. Patients used for the current investigation were enrolled during the first study inclusion period from October 2011 until June 2013, while follow-up lasted until 2015. Patients were recruited during their regular outpatient clinic visit, in the Erasmus MC in Rotterdam or in the Northwest Clinics in Alkmaar. To be eligible for this study, CHF had to be diagnosed ≥3 months ago according to European Society of Cardiology guidelines [12,13]. Also, patients had to be ambulatory and stable, i.e., they should not have been hospitalized for HF in the past three months. The study design of the Bio-SHiFT study (including detailed inclusion and exclusion criteria) has been described in detail previously [14,15]. The study was approved by the medical ethics committees, conducted in accordance with the Declaration of Helsinki, and registered in ClinicalTrials.gov (NCT01851538, https://clinicaltrials.gov/ct2/show/NCT01851538). Written informed consent was obtained from all patients.

### 2.2. Study Procedures

All patients underwent standard care at the outpatient clinic by their treating physicians, who were blinded for biomarker results. Additionally, study follow-up visits were predefined and scheduled every 3 months (±1 month). At the moment of enrolment and at each study follow-up visit, a short medical evaluation was performed, blood samples were collected and occurrence of cardiovascular events since last study visit was recorded. Blood samples were processed and stored at −80 °C within two hours after collection. As biomarkers were measured after completion of follow-up, this information did not lead to change of treatment strategies since treating physicians were unaware of the study results.

### 2.3. Study Endpoints

The primary endpoint (PE) was a composite of cardiac death, heart transplantation, left ventricular assist device implantation, and hospitalization for the management of acute or worsened HF, whichever occurred first. A clinical event committee, blinded for the biomarker results, reviewed hospital records and discharge letters and adjudicated the study endpoints [14,15].

### 2.4. Blood Sample Selection

In this first inclusion period of the Bio-SHiFT study, we collected a total of 1984 samples in 263 patients before occurrence of the PE or censoring (median of 9 (25th–75th percentile: 5–10) blood samples per patient). For reasons of efficiency, we made a selection from these samples: we selected all samples at enrolment, the last sample available in patients in whom the PE did not occur during follow-up, and the two samples available closest in time prior to the PE (which, by design, were 3 months apart). Previous investigations in this cohort have demonstrated that levels of several biomarker change in the months prior to the incident adverse event [14,15]. Thus, by selecting the last two samples prior to the endpoint, we aimed to capture this change. In event-free patients however, our previous investigations showed stable biomarker levels, in which case one additional biomarker sample suffices. In total, this selection amounted to 567 samples for the current analysis.

### 2.5. Biomarker Measurements

To investigate new biomarkers, the cardiovascular panel III of the Olink Multiplex platform (Olink Proteomics AB, Uppsala, Sweden) was used for a batch-wise analysis. This multiplexing assay is based on proximity extension assay technology [16]. The assay uses two oligonucleotide-labelled antibodies to bind to their respective target proteins in the sample. When the two antibodies are in close proximity, a new polymerase chain reaction target sequence is formed. The resulting sequence is detected and quantified using standard real-time PCR. The proteins/biomarkers are delivered in Normalized Protein Expression (NPX) Units, which are relative units that result from the polymerase chain reaction. The NPX units are expressed on a log2 scale where one unit higher NPX represents a doubling of the measured protein concentrations. This arbitrary unit can thus be used for relative quantification of proteins and comparing the fold changes between groups. In the 567 selected samples, we measured C1qR, CDH5, CHI3L1, CNTN1, EPHB4, Ep-CAM, ICAM2, ITGB2, JAM-A, PECAM-1, SELE and SELP. In Appendix A Table A1, an overview is given of the adhesion molecule biomarkers included in this study, including abbreviations, synonyms and function. 

Additionally, in all patients, N-terminal pro–B-type natriuretic peptide (NT-proBNP) and high-sensitive troponin T (hsTnT) were measured using electrochemiluminescence immunoassays (Elecsys 2010; Roche Diagnostics, Indianapolis, IN, USA) as described before [14].

### 2.6. Statistical Analysis

Variables with a normal distribution are presented as the mean ± standard deviation (SD), whereas the median and 25th–75th percentile are used in case of non-normality. Differences between groups were tested with Student t-tests (for normally distributed variables) or with Mann Whitney tests (non-normally distributed variables). Categorical variables were presented as counts and percentages and differences between groups were tested with chi square tests. We used linear mixed effect models to plot the average temporal pattern of each adhesion molecule biomarker for patients with and without a PE during study follow-up.

To estimate the associations between patient-specific repeated biomarker measurements and the hazard of the PE, we applied joint modelling (JM) analyses. JM combines linear mixed effect models for temporal evolution of the repeated measurements with time-to event relative risk models for the time-to-event data [17]. By using the JM technique, analyses inherently accounted for different follow-up durations between patients [18]. We studied the predictive value of biomarker levels, as well as their rates of change (i.e., the slopes of the longitudinal biomarker trajectories). The latter analysis is of particular interest in situations where, for example, at a specific time point two patients show similar marker levels, but differed in rate of change of the marker [19]. First, all JM analyses were performed univariably. Subsequently, we considered a ‘clinical model’ and an ‘established biomarker model’, to adjust for potential confounders. The clinical model was adjusted for age, gender, diabetes mellitus, atrial fibrillation, New York Heart Association (NYHA) class, use of diuretics and systolic blood pressure, while the established cardiac biomarker model was adjusted for NT-proBNP and hsTnT (measured at study enrolment). For all the JM analyses, we used the Z-score (i.e., the standardized form) of the log2-transformed biomarkers to allow for direct comparisons of different biomarkers. Results are given as hazard ratios (HR) with their 95% confidence intervals (CI) per SD change of the biomarker’s level or slope.

We used the conventional *p* < 0.05 threshold to conclude significance for the relation between patient characteristics and the occurrence of the PE during follow-up (Table 1). For the other analyses, we corrected for multiple testing using the Bonferonni correction (*n* = 12), which resulted in a corrected significance level of *p* < 0.004. Analyses were performed with SPSS Statistics 24 (IBM Inc., Chicago, IL, USA) and R Statistical Software using packages nlme [20] and JMbayes [17].

## 3. Results

### 3.1. Baseline Characteristics and Study Endpoints

During a median (25th–75th percentile) follow-up of 2.2 (1.4–2.5) years, a total of 70 (27%) patients reached the PE: 56 patients were re-hospitalized for acute or worsened HF, three patients underwent heart transplantation, two patients underwent left ventricular assistant device implantation, and nine patients died of cardiovascular causes. Table 1 displays the patients’ characteristics at enrolment and the differences in these characteristics between patients who reached the PE during follow-up and patients who did not. The median age was 68 (25th–75th percentile: 59–76), years, with 72% men and 74% NYHA class I–II. The median duration of HF was 4.6 (1.7–9.9) years. Patients who reached the endpoint during follow-up were older and more often in a higher NYHA-class (III or IV), compared to patients who did not reach the PE. They also had a longer duration of HF, lower systolic blood pressures, higher levels of NT-proBNP and hsTNT, were more likely to have atrial fibrillation and diabetes mellitus, and had a higher prevalence of diuretics use. Baseline levels of C1qR, CDH5, CHI3L1, EPHB4 and JAM-A were significantly higher in patients who later experienced the endpoint compared to patients who remained event-free.

### 3.2. Temporal Patterns of Circulating Biomarkers of Cell Adhesion in Relation to Study Endpoints 

**Figure 1:**
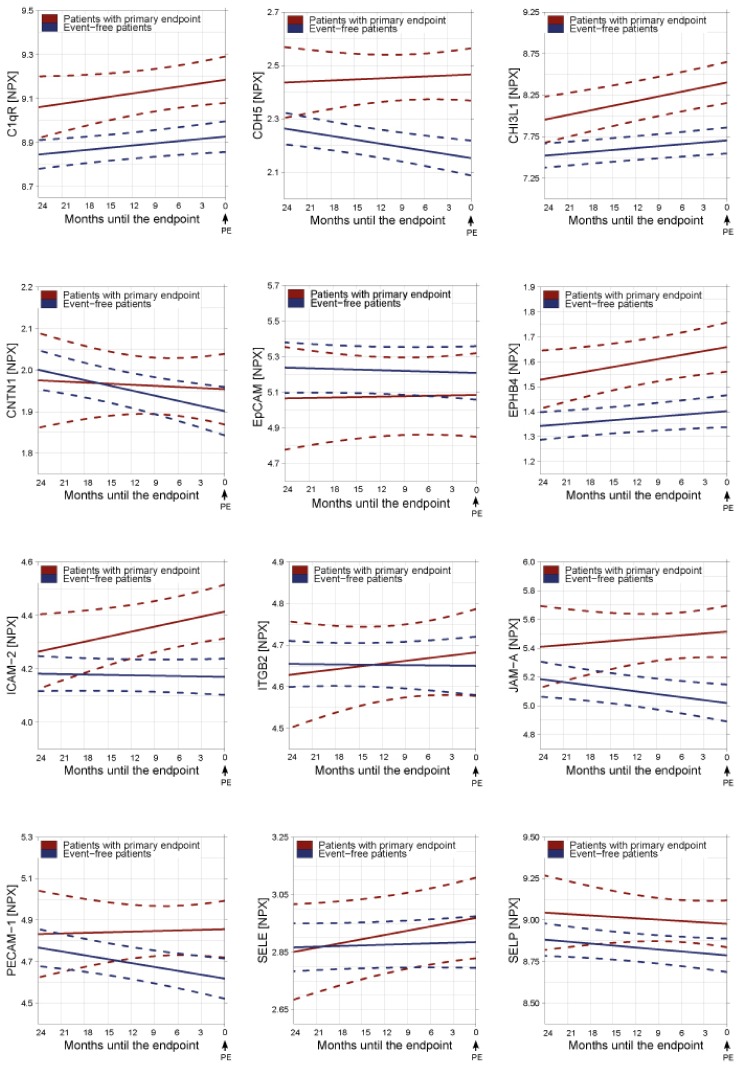
Average temporal patterns of adhesion molecule biomarkers during follow-up approaching the primary endpoint (PE) or last sample moment. *X*-axis: time remaining to the PE (for patients who experienced incident adverse events) or time remaining to last sample moment (for patients who remained event-free). Therefore, ‘time zero’ is defined as the occurrence of the endpoint or last sample moment and is depicted on the right side of the x-axis, so that the average marker trajectory can be visualized as the endpoint approaches. *Y*-axis: biomarker levels in arbitrary, relative units (Normalized Protein Expression, NPX). Solid red line: Average temporal pattern of biomarker levels in patients who reached the primary endpoint during follow-up. Solid blue line: Average temporal pattern of biomarker levels in patients who remained endpoint free (solid blue line). Dashed lines: 95% confidence interval. Abbreviations: Complement component C1q receptor: C1qR, Cadherin 5: CDH5, Chitinase-3-like protein 1: CHI3L1, CNTN1: Contactin-1, Ep-CAM: Epithelial cell adhesion molecule, EPHB4: Ephrin type-B receptor 4, ICAM2: Intercellular adhesion molecule-2, ITGB2: Integrin beta-2, JAM-A: Junctional adhesion molecule A, NPX: Normalized Protein Expression, PE: primary endpoint, PECAM-1: Platelet endothelial cell adhesion molecule 1, SELE: E-selectin, and SELP: P-selectin.

Figure 1 depicts the average temporal evolutions of biomarkers of cell adhesion from twenty-four months before the PE or before last sample moment (for patients who remained event-free) onwards, based on linear mixed effect models. As the endpoint or last sample moment approached, biomarkers C1qR, CDH5, CHI3L1, EPHB4, ICAM-2 and JAM-A showed higher levels in patients who experienced the PE versus those who remained event-free. Some were already higher 24 months before the endpoint, while others were not but diverged as the end-point drew closer. On the other hand, CNTN1, EpCAM, ITGB2, PECAM-1, SELE and SELP did not show a clear difference between both groups.

Table 2 shows the associations of the repeatedly measured levels of biomarkers of cell adhesion with the PE based on JM analyses. C1qR showed the strongest association in univariate analysis with a HR of 2.22 (95% CI: 1.62–3.10) per SD change at any point in time during follow-up. After adjustment for clinical characteristics, CHI3L1 remained the strongest predictor of the PE, with a HR of 2.27 (95% CI: 1.66–3.16). CHI3L1 was followed by JAM-A (HR 2.10, 95% CI: 1.42–3.23) and C1qR (HR 1.90, 95% CI: 1.36–2.72). In addition, the risk estimates of CHI3L1 (HR 1.68, 95% CI: 1.23–2.35) and JAM-A (HR 1.75, 95% CI: 1.25–2.49) remained significant after adjustment for baseline established cardiac biomarkers NT-proBNP and hsTNT.

Apart from evaluating the predictive value of repeatedly assessed biomarker levels, we also evaluated their rates of change (i.e., the slopes of the longitudinal biomarker trajectories) and concurrent HRs. Although the trajectories plotted by using linear mixed effect models (Figure 1) have already provided an impression of temporal evolution of biomarker level in those with and without incident PEs, evaluating slope by means of the JM provides the possibility to evaluate instantaneous slope, which may render additional insights. In these analyses, the same biomarkers remained significant predictors of the PE, i.e., CDH5, CD93, CHI3L1, EPHB4, ICAM-2 and JAM-A, even after adjusting for clinical factors (Table 3). JAM-A showed numerically the strongest association with the PE with a HR of 1.64 (95% CI: 1.23–2.24) per 0.1SD change of the annual slope, followed by CHI3L1 (HR 1.58, 95% CI: 1.36–1.93) and CDH5 (HR 1.47, 95% CI: 1.17–2.00).

## 4. Discussion

In the present study, we found that biomarkers of cell adhesion C1qR, CDH5, CHI3L1, EPHB4, ICAM-2 and JAM-A were associated with clinical outcomes in 263 stable patients with CHF. At baseline, levels of biomarkers C1qR, CDH5, CHI3L1, EPHB4 and JAM-A were higher in patients who later experienced the PE compared to patients who remained event-free. Furthermore, the average biomarker evolutions over time of these markers, and additionally of ICAM-2, showed higher levels as the PE approached. Even more important, repeatedly measured levels of these biomarkers of cell adhesion were independently associated with the PE. Even adjusted for clinical factors, biomarkers of cell adhesion served as predictors of clinical adverse events.

Recent studies suggest a pivotal role of CAMs in the processes of HF. Until now, however, research on CAMs in relation to adverse clinical outcomes in patients with CHF is limited. Previous studies have mostly described the value of single measurements of adhesion molecules (e.g., at admission) for prognosis, and studies were relatively small. Our study, which was based on repeated measurements, demonstrates a promising role for several adhesion biomarkers for individual prognostication in CHF patients Temporal patterns shortly before an adverse event occurs have not yet been investigated in detail previously, while this might be a crucial time window for therapeutic interventions.

In our study, CHI3L1 was the biomarker whose association with the PE was numerically the strongest after adjustment for clinical factors. CHI3L1 is a glycoprotein secreted in vitro by cells such as activated macrophages and neutrophils in different tissues with inflammation. Studies on patients with acute myocardial infarction, stable coronary artery disease, atrial fibrillation and CHF have demonstrated elevated levels of CHI3L1 compared with healthy controls [21]. Moreover, several studies have previously examined CHI3L1 in relation to clinical outcome in CHF; but repeated measurements were never used. Some of these studies showed that CHI3L1 is associated with all-cause mortality [22] and that it is able to detect patients at high risk for adverse outcomes as well [23,24]. Other studies failed to demonstrate such associations. Rathcke at al. examined CHI3L1 levels in patients with CHF and in age-matched controls without cardiovascular disease [25]. They found higher levels of CHI3L1 at baseline in patients with CHF, but these levels did not predict cardiovascular events or overall mortality. Mathiasen et al. [21] suggested that, most likely, elevated levels of CHI3L1 in CHF patients are explained by the presence of concomitant diseases. CHF is a complex disorder, often complicated by other comorbidities in which CHI3L1 is known to be elevated, such as arrhythmias, renal dysfunction, diabetes mellitus and hypertension. These concomitant diseases could thus possibly explain the differences in CHI3L1 levels when compared to healthy individuals. Conversely, in our study, we not only adjusted for age, but also for clinical factors, and still we found an association between CHI3L1 and clinical adverse events.

The barrier formed by endothelial cells allows regulated passage of immune cells in the normal state and during inflammatory conditions. This passage is mediated through junctional molecules, such as ICAM-2, CDH5, JAMA, and PECAM-1 [26,27]. ICAM-2 participates in the docking of leukocytes to the endothelium, and is likely to be relevant for leukocyte diapedesis [28]. For example, former research showed that endothelial cell activation leads to neutrophil transmigration, supported by the sequential roles of ICAM-2, JAM-A and PECAM-1 [26]. We are not aware of previous investigations that link ICAM-2 to prognosis of stable CHF patients. We show that rate of change of ICAM-2 independently predicts adverse clinical outcome. This suggests that prognosis differs between patients with stable ICAM-2 values and patients with increasing ICAM-2 values. CDH5 is an endothelial transmembrane glycoprotein and is the major molecule for cell–cell adhesion that forms adherens junctions [9]. Shedding of CDH5 into the circulation is associated with severe acute kidney injury and with more severe organ dysfunction in patients with sepsis [29] and increased levels of soluble CDH5 were associated with poor outcome in severe sepsis [30]. In cardiovascular research, elevated levels of CDH5 have also been reported to be associated with coronary atherosclerosis [31]. Based on our results, CDH5 may be of use as a biomarker that reflects on-going inflammation and indicates impending adverse events in CHF patients. JAM-A is involved in the regulation of vascular permeability [27] and genetic deletion and blockade of JAM-A generally results in increased permeability of endothelial cells [32]. JAM-A is also thought to be required for movement of leukocytes toward sites of inflammation [33] and it may be considered as a marker of acute endothelial activation and dysfunction [34]. This is in line with our findings; we demonstrate that repeatedly measured levels of JAM-A show a numerically strong independent association with the PE. The significant role of PECAM-1 in platelet aggregation and migration of leukocytes through the endothelium [35] is interesting in the context of CHF. PECAM-1 has been suggested as a sensitive marker providing early diagnostic aid in acute coronary syndromes [36]. In heart failure research, soluble PECAM-1 was found to be elevated in the majority of patients with severe CHF [37]. However, we did not find an association of PECAM-1 with prognosis in our CHF cohort.

SELP is of great interest because of its key role in interactions between platelets, leucocytes, and endothelium [38]. Abnormal surface SELP expression [39,40] and soluble SELP levels [41] have been reported in decompensated heart failure, suggesting persistent platelet activation. Regarding their prognostic value, however, levels of soluble SELP, platelet surface SELP, and total platelet SELP did not determine prognosis [42] and our results support these findings. Ep-CAM, CNTN1, ITGB2, and SELE also showed negative results in our study. 

Less is known about the other biomarkers in relation to CHF. For example, C1qR is a transmembrane receptor once thought to be only a receptor for C1q, but is now thought to play a role in endothelial cell adhesion [43]. The up-regulation of this receptor by inflammatory mediators and the ability of complement component C1q itself to increase ICAM-1 expression suggest a potential role for the receptor in vascular inflammation and immune injury [44]. To the best of our knowledge, C1qR has never been linked directly to prognostication in CHF patients. In our study, repeatedly measured levels of this marker were independently associated with the PE. EPHB4 serves as receptor for its transmembrane ligand ephrin-B2. Both are specifically expressed on arterial and venous endothelial cells. Hamada et al. concluded ephrin-B2 forward signaling and EPHB4 reverse signaling differentially affect cell adhesion and migration between arterial and venous endothelial cells [45]. We found that both level and slope analysis of EPHB4 were significantly associated with the endpoint, even after adjusting for clinical factors.

While the 263 patients included in our investigation were ambulatory and stable, it has been advocated that grouping of HF patients should not be approached only based on symptoms [46], nor on ejection fraction solely [47]. Definitions have been described to identify more advanced disease HF (AdHF), i.e., patients with worsening clinical condition, high rates of re-hospitalization and mortality (meaning a condition where standard treatments are inadequate and additional interventions must be applied; these patients are suitable for LVAD), as well as end-stage heart failure (patients for which advanced therapies, such as LVAD, is contraindicated and palliative cares should be pursued) [48]. In post-hoc analyses, based on our available data, we identified at least 57 patients who might be categorized into these two groups at baseline; given their ambulant condition most likely the AdHF group. Thirty of them eventually experienced an endpoint during follow-up. Compared to the other 206 patients, these 57 patients were older, had a higher heart rate, lower systolic and diastolic blood pressure, had higher NT-proBNP, hsTnT and eGFR levels, and were more likely to have prior CVA/TIA and diabetes mellitus. Malfunction of other organs could affect prognosis [49], and, therefore, differences in such risk factors should be taken into account (as for example also highlighted in a recent study about the role of oxidative stress and vascular inflammation in diabetic patients which could result in myocardial infarction [50]). Since we adjusted our current analyses of the association between circulating biomarkers of cell adhesion and clinical outcomes for variables such as diabetes mellitus and atrial fibrillation, we believe we have accounted for this type of confounding as much as we could in this observational study.

Our study has some limitations. First, because of efficiency reasons, we did not use all 1984 available trimonthly samples, but selected 3 samples for patients with a PE (baseline and last 2 prior to the PE), and 2 samples for event-free patients, resulting in 567 samples. Our previous investigations using all samples demonstrated that most of the examined biomarkers show an increase shortly prior to the incident adverse event. Thus, we believe that with our approach we retain the most informative measurements while enhancing efficiency. Second, as described before [15,51], our cohort comprised mainly HF patients with a reduced ejection fraction. This can most likely be attributed to the fact that in the Netherlands, most HF patients with a preserved ejection fraction are treated in secondary referral centers or by the general practitioner. Finally, we used biomarker values in Normalized Protein Expression (NPX) Units, i.e., relative units. While these values can be used for comparing patients and changes over time within a patient, for clinical applications absolute concentrations are recommended. 

In conclusion, the present study demonstrates that serial measurements of C1qR, CDH5, CHI3L1, EPHB4, ICAM-2 and JAM-A are independently associated with clinical adverse events in patients with CHF, suggesting that markers of cell adhesion could be useful for individual risk profiling. These biomarkers are also interesting for future therapeutic purposes, as CAMs may be used as targets to inhibit vascular inflammation and endothelial dysfunction. Further studies are warranted to confirm these associations, to investigate whether a combination of different markers (for example C1qR, CHI3L1 and JAM-A) may improve prognostication and to better elucidate the pathophysiological role of cell adhesion in CHF. 

## Figures and Tables

**Table 1:** Patients characteristics in relation to the occurrence of the primary endpoint (PE).

Variable	Total	PE Reached during Follow-Up		*p*-Value
		**Yes**	**No**	
	263 (100)	70 (27)	193 (73)	
Demographics				
Age—years	68 (59–76)	72 (60–80)	67 (58–75)	0.021 *
Men	189 (72)	53 (76)	136 (71)	0.40
Clinical characteristics				
Body Mass Index (kg/m^2^)	26 (24–30)	27 (24–30)	26 (24–30)	0.80
Heart rate (eats/min)	67 ± 12	69 ± 13	67 ± 11	0.22
Systolic blood pressure (mmHg)	122 ± 20	117 ± 17	124 ± 21	0.020 *
Diastolic blood pressure (mmHg)	72 ± 11	70 ± 10	73 ± 11	0.06
Features of heart failure				
Duration of HF (years)	4.6 (1.7–9.9)	6.8 (2.8–12.5)	3.8 (1.1–8.2)	0.002 *
NYHA class III or IV	69 (26)	31 (44)	38 (20)	<0.001 *
HF with reduced ejection fraction	250 (95)	66 (94)	184 (95)	0.75
HF with preserved ejection fraction	13 (5)	4 (6)	9 (5)	
Left ventricular ejection fraction	31 ± 11	28 ± 11	31 ± 11	0.108
Established biomarkers				
NT-proBNP (pmol/L)	137 (52–273)	282 (176–517)	95 (32–208)	<0.001 *
HsTnT (ng/L)	18 (10–33)	32 (21–50)	14 (8–27)	<0.001 *
eGFR (mL/min per 1.73m^2^)	58 (43–76)	53 (40–73)	59 (44–77)	0.20
Etiology of heart failure				
Ischemic	117 (45)	36 (51)	81 (42)	0.17
Hypertension	34 (13)	10 (14)	24 (12)	0.69
Secondary to valvular disease	12 (5)	5 (7)	7 (4)	0.31
Cardiomyopathy	68 (26)	15 (21)	53 (28)	0.32
Unknown or Others	32 (12)	4 (6)	28 (15)	
Medical history				
Prior Myocardial infarction	96 (37)	32 (46)	64 (33)	0.060
Prior Percutaneous coronary intervention	82 (31)	27 (39)	55 (29)	0.12
Prior Coronary artery bypass grafting	43 (16)	13 (19)	30 (16)	0.56
Prior CVA/TIA	42 (16)	15 (21)	27 (14)	0.15
Atrial fibrillation	106 (40)	36 (51)	70 (36)	0.027 *
Diabetes Mellitus	81 (31)	32 (46)	49 (25)	0.002 *
Hypercholesterolemia	96 (37)	30 (43)	66 (34)	0.20
Hypertension	120 (46)	38 (54)	82 (43)	0.090
COPD	31 (12)	12 (17)	19 (10)	0.11
Medication use				
Beta-blocker	236 (90)	61 (87)	175 (91)	0.40
ACE-I or ARB	245 (93)	63 (90)	182 (94)	0.22
Diuretics	237 (90)	68 (97)	169 (88)	0.021 *
Loop diuretics	236 (90)	68 (97)	168 (87)	0.017 *
Thiazides	7 (3)	3 (4)	4 (2)	0.39
Aldosterone antagonist	179 (68)	53 (76)	126 (65)	0.11
Biomarker level at baseline in arbitrary unit (NPX values)				
C1qR	8.88 (8.56–9.27)	9.16 (8.78–9.50)	8.78 (8.50–9.20)	<0.001 *
CDH5	2.29 (2.00–2.67)	2.36 (2.12–2.84)	2.27 (1.96–2.60)	0.010 *
CHI3L1	7.68 (6.88–8.39)	8.08 (7.53–8.72)	7.47 (6.68–8.20)	<0.001 *
CNTN1	2.01 (1.72–2.25)	2.00 (1.68–2.22)	2.01 (1.75–2.27)	0.58
EpCAM	5.11 (4.38–5.82)	4.91 (4.40–5.71)	5.18 (4.36–5.90)	0.41
EPHB4	1.35 (1.08–1.66)	1.55 (1.19–1.95)	1.31 (1.05–1.58)	<0.001 *
ICAM-2	4.20 (3.88–4.59)	4.35 (4.00–4.64)	4.18 (3.85–4.51)	0.061
ITGB2	4.65 (4.39–4.90)	4.64 (4.41–4.96)	4.67 (4.39–4.89)	0.86
JAM-A	5.22 (4.64–5.80)	5.41 (4.79–6.02)	5.08 (4.56–5.71)	0.024 *
PECAM-1	4.74 (4.36–5.17)	4.77 (4.36–5.39)	4.70 (4.35–5.10)	0.32
SELE	2.89 (2.46–3.28)	3.06 (2.51–3.32)	2.84 (2.45–3.28)	0.40
SELP	8.84 (8.46–9.38)	8.98 (8.54–9.58)	8.78 (8.42–9.28)	0.087

Variables with a normal distribution are presented as the mean ± SD, whereas non-normally distributed continuous variables are expressed as the median (25th–75th percentile). Categorical variables are expressed as counts (percentages). Missing values < 5% if applicable, except for systolic blood pressure (5.3%). * *p*-value < 0.05. ACE-I: angiotensin-converting enzyme inhibitors, ARB: angiotensin II receptor blockers, C1qR: complement component C1q receptor, CDH5: cadherin 5, CHI3L1: chitinase-3-like protein 1, CNTN1: contactin-1, COPD: chronic obstructive pulmonary disease, CVA: cerebrovascular accident, eGFR: estimated glomerular filtration rate, Ep-CAM: epithelial cell adhesion molecule, EPHB4: Ephrin type-B receptor 4, HF: heart failure, HsTnT: high-sensitive troponin T, ICAM-2: intercellular adhesion molecule-2, ITGB2: integrin beta-2, JAMA: junctional adhesion molecule A, NPX, Normalized Protein Expression, NT-proBNP: N-terminal pro–B-type natriuretic peptide, NYHA: New York Heart Association, PECAM-1: Platelet endothelial cell adhesion molecule 1, SELE: E-selectin, SELP: P-selectin and TIA: transitory ischemic attack.

**Table 2:** Associations between the levels of biomarkers of cell adhesion and the primary endpoint.

	Crude Model		Clinical Model		Biomarker Model	
Biomarker	HR (95% CI)	*p*-Value	HR (95% CI)	*p*-Value	HR (95% CI)	*p*-Value
C1qR	2.22 (1.62–3.10)	<0.001 *	1.90 (1.36–2.72)	<0.001 *	1.47 (1.04–2.14)	0.028
CDH5	2.01 (1.47–2.77)	<0.001 *	1.79 (1.30–2.50)	<0.001 *	1.56 (1.14–2.14)	0.004
CHI3L1	2.11 (1.60–2.84)	<0.001 *	2.27 (1.66–3.16)	<0.001 *	1.68 (1.23–2.35)	0.002 *
CNTN1	0.93 (0.66–1.32)	0.70	0.98 (0.67–1.45)	0.92	0.93 (0.66–1.31)	0.66
EpCAM	0.86 (0.66–1.11)	0.27	0.90 (0.67–1.20)	0.46	0.90 (0.69–1.17)	0.46
EPHB4	1.90 (1.48–2.44)	<0.001 *	1.77 (1.35–2.33)	<0.001 *	1.37 (1.03–1.80)	0.031
ICAM2	2.08 (1.51–2.94)	<0.001 *	1.79 (1.29–2.53)	0.001 *	1.53 (1.12–2.12)	0.005
ITGB2	1.07 (0.77–1.47)	0.70	0.95 (0.65–1.37)	0.77	1.04 (0.75–1.42)	0.83
JAM-A	1.86 (1.34–2.63)	<0.001 *	2.10 (1.42–3.23)	<0.001 *	1.75 (1.25–2.49)	0.001 *
PECAM-1	1.39 (1.00–1.94)	0.050	1.60 (1.10–2.35)	0.013	1.47 (1.04–2.08)	0.031
SELE	1.11 (0.86–1.44)	0.43	1.07 (0.81–1.40)	0,66	1.11 (0.86–1.44)	0.43
SELP	1.34 (0.98–1.86)	0.071	1.45 (1.01–2.10)	0.044	1.49 (1.08–2.06)	0.018

Hazard ratios (HRs) and 95% confidence intervals (CIs) are given per standard deviation change at any point in time during follow-up, which were estimated by joint modelling (JM) analysis. JM combines linear mixed effect (LME) models for the temporal evolution of the repeated measurements with Cox proportional hazard models for the time-to-event data. Thus, all available measurements are simultaneously taken into account in the current analyses (i.e., all baseline samples, the last sample available in patients in whom the PE did not occur during follow-up, and the two samples available closest in time prior to the primary endpoint). Crude model: Cox model unadjusted, LME model unadjusted; Clinical model: Cox and LME models adjusted for age, sex, diabetes, atrial fibrillation, baseline New York Heart Association class, diuretics, and systolic blood pressure; Established cardiac biomarker model: Cox and LME models adjusted for baseline NT-proBNP and hsTnT. Data for systolic blood pressure was missing in >5% of patients. Imputations were applied using the patients’ clinical and outcome data. * *p*-value below the corrected significance level for multiple testing (*p*-value < 0.004).

**Table 3:** Associations between the slope of biomarkers of cell adhesion and the primary endpoint.

	Crude Model		Clinical Model		Biomarker Model	
Biomarker	HR (95% CI)	*p*-Value	HR (95% CI)	*p*-Value	HR (95% CI)	*p*-Value
C1qR	1.34 (1.16–1.56)	<0.001 *	1.43 (1.13–1.92)	0.002 *	1.12 (1.02–1.24)	0.019
CDH5	1.36 (1.18–1.60)	<0.001 *	1.47 (1.17–2.00)	<0.001 *	1.16 (1.07–1.27)	<0.001 *
CHI3L1	1.41 (1.29–1.57)	<0.001 *	1.58 (1.36–1.93)	<0.001 *	1.27 (1.18–1.39)	<0.001 *
CNTN1	1.04 (0.94–1.17)	0.45	1.04 (0.92–1.18)	0.53	1.06 (0.98–1.15)	0.13
EpCAM	1.01 (0.88–1.16)	0.92	1.01 (0.88–1.17)	0.88	1.01 (0.92–1.11)	0.83
EPHB4	1.33 (1.19–1.51)	<0.001 *	1.34 (1.15–1.68)	<0.001 *	1.14 (1.04–1.25)	0.005
ICAM2	1.32 (1.22–1.45)	<0.001 *	1.44 (1.27–1.72)	<0.001 *	1.22 (1.15–1.31)	<0.001 *
ITGB2	1.07 (0.94–1.21)	0.32	0.99 (0.83–1.16)	0.90	1.05 (0.97–1.15)	0.23
JAM-A	1.34 (1.12–1.62)	0.002 *	1.64 (1.23–2.24)	0.001 *	1.10 (0.99–1.24)	0.085
PECAM-1	1.15 (0.98–1.40)	0.088	1.09 (0.86–1.72)	0.80	1.06 (0.97–1.18)	0.21
SELE	1.21 (1.05–1.41)	0.015	1.19 (0.99–1.41)	0.060	1.10 (0.96–1.23)	0.15
SELP	1.29 (1.13–1.49)	0.020	1.45 (1.22–1.84)	<0.001 *	1.12 (0.94–1.27)	0.15

Hazard ratios (HRs) and 95% confidence intervals (CIs) are given per 0.1 standard deviation of the annual slope at any point in time during follow-up, which were estimated by joint modelling (JM) analysis. JM combines linear mixed effect (LME) models for the temporal evolution of the repeated measurements with Cox proportional hazard models for the time-to-event data. Thus, all available measurements are simultaneously taken into account in the current analyses (i.e., all baseline samples, the last sample available in patients in whom the PE did not occur during follow-up, and the two samples available closest in time prior to the primary endpoint). Crude model: Cox model unadjusted, LME model unadjusted; Clinical model: Cox and LME models adjusted for age, sex, diabetes, atrial fibrillation, baseline New York Heart Association class, diuretics and systolic blood pressure; Established cardiac biomarker model: Cox and LME models adjusted for baseline NT-proBNP and hsTnT. Data for systolic blood pressure was missing in >5% of patients. Imputations were applied using the patients’ clinical and outcome data. * *p*-value below the corrected significance level for multiple testing (*p*-value < 0.004).

## Appendix A

**Table A1:** Overview of the assessed biomarkers of cell adhesion.

Abbreviation	Full Name	Synonyms	Function
C1qR	Complement component C1q receptor	CD93	Stimulates endothelial expression of adhesion molecules/C1q-mediated endothelial cell adhesion
CDH5	Cadherin 5	VE cadherin	Major cell–cell adhesion molecule that forms adherens junctions
CHI3L1	Chitinase-3-like protein 1	YKL-40, HC gp39, brp-39, gp38k, and MGP-40	Endothelial activation and dysfunction
CNTN1	Contactin-1	GP130	Expressed in neuronal tissues, associates with other cell surface proteins and believed to participate in signal transduction pathways and cell functions
Ep-CAM	Epithelial cell adhesion molecule	CD326	Cell–cell adhesion molecule and part of diverse processes such as signaling, cell migration, proliferation, and differentiation
EPHB4	Ephrin type-B receptor 4	HTK and Tyro11	Essential role in vascular development
ICAM-2	Intercellular adhesion molecule-2	CD102	Adherence and transmigration of leucocytes
ITGB2	Integrin beta-2	CD18	Ligands for ICAM-1, and critical for the migration of leucocytes to sites of inflammation
JAM-A	Junctional adhesion molecule A	F11R	Involved in the migration of leukocytes through the endothelial cell barrier
PECAM-1	Platelet endothelial cell adhesion molecule 1	CD31	Platelet/endothelial interaction, adherence and transmigration of leucocytes
SELE	E-selectin	CD62E, ELAM-1, and LECAM2	Leucocyte rolling
SELP	P-selectin	CD154	Platelet/endothelial interaction and leucocyte rolling
